# *Ascaridia hermaphrodita* (Froelich, 1789) and *Ascaridia columbae* (Gmelin, 1780) in neotropical psittacine birds

**DOI:** 10.1590/S1984-29612025015

**Published:** 2025-04-18

**Authors:** Octávio Augusto Serra Santos, Vanessa Kanaan, Hugo Lago de Sant’Anna Rocha, Reinaldo José́ da Silva, Tânia Freitas Raso

**Affiliations:** 1 Departamento de Patologia Veterinária, Faculdade de Medicina Veterinária e Zootecnia, Universidade de São Paulo – USP, São Paulo, SP, Brasil; 2 Centro de Triagem de Animais Silvestres de Santa Catarina, Instituto Espaço Silvestre, Florianópolis, SC, Brasil; 3 Setor de Parasitologia, Instituto de Biociências, Universidade Estadual Paulista – UNESP, Botucatu, SP, Brasil

**Keywords:** Ascariasis, wild birds, scanning electron microscopy, parasitology, Ascaridíase, aves silvestres, microscopia eletrônica de varredura, parasitologia

## Abstract

Gastrointestinal parasites found in four Neotropical psittacine birds of the species *Ara macao*, *Amazona aestiva, Amazona vinacea* and *Pionus maximiliani* have been reported. The carcasses of the animals were received from commercial breeders and a rehabilitation center for necropsy. In total, 589 parasites were collected during the exams and submitted for morphological analysis and taxonomic identification. Twenty parasites from each host were cleaned with lactophenol and analyzed by light microscopy, while four parasites from each host were prepared for scanning electron microscopy. Tissue samples were forwarded for histological analysis in search of erratic larvae; however, none were found. Parasites from *A. macao, A. aestiva* and *A. vinacea* were identified as *Ascaridia hermaphrodita*, whereas parasites from *P. maximiliani* were identified as *Ascaridia columbae*, promoting the first report of *A. columbae* in *P. maximiliani.* All birds were kept in enclosures with access to the ground, facilitating parasitism. *Pionus maximiliani* and *A. vinacea* were kept together in the same enclosure with high population density. Additionally, synanthropic animals, such as Columbiformes, were observed in the same enclosure, facilitating infection with *A. columbae.* The identification of *Ascaridia* species that parasitizes psittacine birds helps to improve prevention and control measures, thus enhancing avian health and welfare.

## Resumo

Parasitas gastrointestinais encontrados em quatro psitacídeos neotropicais das espécies *Ara macao*, *Amazona aestiva, Amazona vinacea* e *Pionus maximiliani* foram relatados. As carcaças dos animais foram recebidas para necropsia de criatórios comerciais e de um centro de reabilitação. Ao todo, coletaram-se 589 parasitas durante os exames e os submeteram à análise morfológica e à identificação taxonômica. Vinte parasitas de cada hospedeiro foram clarificados com lactofenol e analisados por microscopia de luz, enquanto quatro parasitas de cada hospedeiro foram preparados para microscopia eletrônica de varredura. Analisaram-se amostras de tecido histologicamente em busca de larvas erráticas, mas nenhuma foi encontrada. Parasitas de *A. macao, A. aestiva* e *A. vinacea* foram identificados como *Ascaridia hermaphrodita*, enquanto parasitas oriundos do *P. maximiliani* identificados como *Ascaridia columbae*, sendo esse, o primeiro relato de *A. columbae* em *P. maximiliani.* Todas as aves eram mantidas em recintos com acesso ao solo, favorecendo o parasitismo. *Pionus maximiliani* e *A. vinacea* eram mantidos juntos em recinto com alta densidade populacional. Além disso, observaram-se animais sinantrópicos, como Columbiformes, nesse recinto, facilitando a infecção por *A. columbae*. A identificação de espécies de *Ascaridia* que parasitam psitacídeos colabora para aprimorar medidas de prevenção e controle, aumentando assim a saúde e bem-estar das aves.

## Introduction

Nematode parasites of the genus *Ascaridia* (Dujardin, 1845) have previously been detected in the order Psittaciformes worldwide, with eight species reported (Kajerová et al., 2004; Abe et al., 2015). *Ascaridia hermaphrodita* (Froelich, 1789) and *Ascaridia sergiomeirai* (Pereira, 1933) are commonly found in Neotropical parrots; however, the former is more significant due to its higher frequency in cases of ascariasis (Pinto et al., 1993; Melo et al., 2013). *Ascaridia ornata* (Kreis, 1955) and *Ascaridia nicobarensis* (Soota et al., 1970) are known for their first and only reports in *Amazona amazonica* (Linnaeus, 1766) and *Psittacula longicauda nicobarica* (Gould, 1857), respectively (Kajerová et al., 2004; Soota et al., 1970). *Ascaridia platyceri* (Hartwich & Tscherner, 1979) affects psittacine birds in Australia, whereas *Ascaridia nymphii* (Abe et al., 2015) has been reported in *Nymphicus hollandicus* (Kerr, 1792) and *Ara chloroptera* (Gray, 1859) in Japan and China (Kajerová et al., 2004; Abe et al., 2015; Yang et al., 2018). Although *Ascaridia galli* (Schrank, 1788) and *Ascaridia columbae* (Gmelin, 1780) have been reported in psittacine birds, they normally parasitize fowl and pigeons, respectively (Mines & Green, 1983; Patel et al., 2000).

Parasitized birds often do not present clinical manifestations. However, at high parasite loads, clinical signs include lethargy, weight loss, anorexia, diarrhea, hematochezia, and ataxia. In severe cases, infection can lead to intestinal obstruction and death (Wilson et al., 1999; Barros et al., 2002; Melo et al., 2013; Grespan & Raso, 2014; Abe et al., 2015).

The life cycle of ascarids is direct. The ingestion of food, water, or feces contaminated with parasite eggs is the main route of transmission. After ingestion, the larvae hatch and migrate into the mucosa of the small intestine, returning to the lumen for maturation and elimination of the eggs (Wehr & Hwang, 1964). Occasionally, erratic migration of larvae from some species may occur within the liver and biliary ducts (Wehr & Hwang, 1964; Mines & Green, 1983; Wilson et al., 1999).

Understanding the distribution, occurrence, and diversity of *Ascaridia* spp. in psittacine birds not only contributes to the knowledge of avian parasitology but also contributes to avian welfare. Therefore, this study aims to report the intestinal parasites of four adult Brazilian psittacine birds kept in captivity and correlate them with management implications.

## Material and Methods

### Necroscopic examination and sample collection

The carcasses were received during the laboratory routine as follows: *Ara macao* (Linnaeus, 1758) and *Amazona aestiva* (Linnaeus, 1758) from two separate avian breeders, as well as *Amazona vinacea* (Kuhl, 1820) and *Pionus maximiliani* (Kuhl, 1820) from a rehabilitation center. All specimens were subjected to necropsy, and tissue samples from *A. macao*, *A. vinacea,* and *P. maximiliani* were collected for histopathological analysis to identify any erratic larvae. The samples were fixed in 10% formaldehyde solution, dehydrated in alcohol, cleared in xylene, and embedded in paraffin blocks. The blocks were cut using a microtome and stained with hematoxylin and eosin (HE) for histological analysis by light microscopy.

### Parasitological analysis

Parasites were collected from all birds for taxonomic identification and kept in 70% ethanol for 48 hours. Ten males and ten females, from each host (except from *A. vinacea*), were subsequently cleared with lactophenol for morphological analysis and taxonomical identification according to Vicente et al. (1995) and Kajerová et al. (2004). The parasites were analyzed using a computerized system for image analysis with differential interference contrast (DIC) - LAS V3 (Leica Application Suite V3; Leica Microsystems, Wetzlar, Germany).

Two males and two females from each host were analyzed using scanning electron microscopy (SEM). For this purpose, nematodes were selected, washed, transferred to 2.5% glutaraldehyde, dehydrated in a graded (30%, 50%, 70%, and 96% ethanol series), dried, and mounted on a strip of conductive carbon tape. The samples were sputter coated with gold and observed at 10 kV in a Hitachi Stereoscan Model SU1510 (Hitachi Ltd., Tokyo, Japan).

## Results

The carcass of the *A. macao* (female, 890g) exhibited a body condition score 2/5 and poor feather condition. The liver was enlarged with pale areas all over the surface. The proventriculus was discretely dilated, and the intestine was hyperemic, with an abundant number of nematodes obstructing the lumen in some segments; 321 parasites were collected.

**Figure 1:**
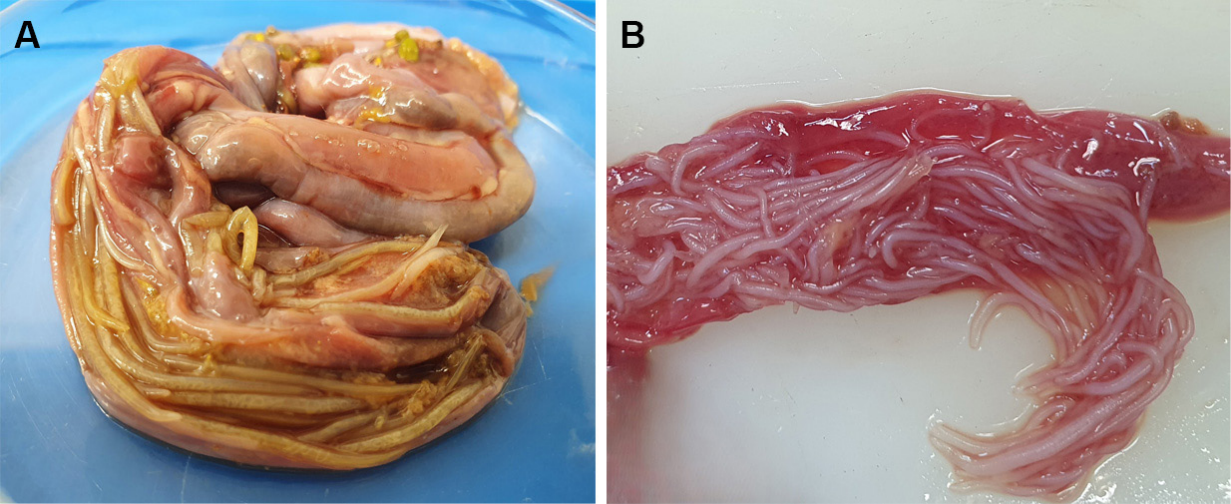
Nematodes found in the intestine of captive psittacines: (A) *Amazona aestiva*; (B) *Pionus maximiliani*.

The *A. aestiva* specimen (male, 405 g) had a body condition score of 2/5. The intestinal mucosa was thickened with the presence of mucous content. Additionally, nematodes were present throughout the entire lumen, and 95 parasites were collected (Figure 1A).

The *A. vinacea* specimen (male, 375 g) presented a body condition score of 3/5 and pales areas in both the liver capsule and parenchyma. Despite the digestive tract being at the beginning of autolysis, 13 nematodes were collected from the intestinal lumen.

The *P. maximiliani* specimen (female, 267 g) presented a body condition score of 3/5, and no significant macroscopic changes were observed. However, 160 parasites were collected from the intestinal lumen (Figure 1B). Throughout the histological analysis, no erratic larvae were found in any tissue sample from the animals necropsied.

**Figure 2:**
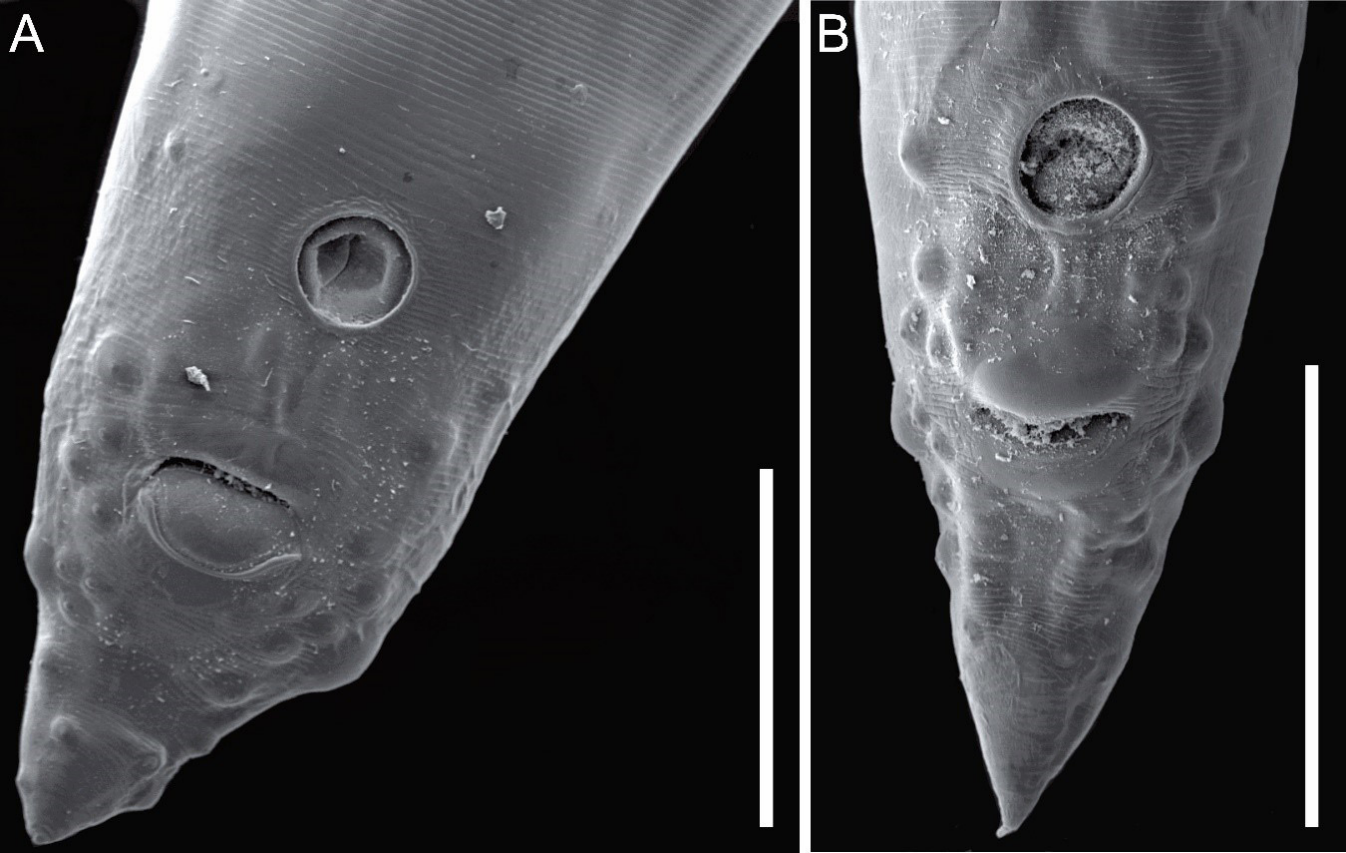
(A) Posterior end of a male of *Ascaridia hermaphrodita* (Froelich, 1789) found in the intestine of *Amazona aestiva* (Linnaeus, 1758), scale bar 500 mm; (B) Posterior end of a male of *Ascaridia columbae* (Gmelin, 1790) found in the intestine of *Pionus maximiliani* (Kuhl, 1820), scale bar 400 mm.

Nematodes derived from *A. macao, A. aestiva,* and *A. vinacea* were identified as *Ascaridia hermaphrodita* (Froelich, 1789) (Figure 2A), whereas parasites from *P. maximiliani* were identified as *Ascaridia columbae* (Gmelin, 1790) (Figure 2B).

*Ascaridia hermaphrodita* has a mouth with three lips, without interlabia, a club-shaped esophagus lacking the posterior bulb, and lateral wings are usually present. Female *A. hermaphrodita* specimens have a vulva close to the middle of the body and a uterus with divergent rami and their eggs have a thick shell. Males have weakly developed caudal wings and subequal spicules and lack a gubernaculum. Males also have a protruding precloacal sucker, with thickened edges. Posterior regions of *A. hermaphrodita* have 15–16 large caudal papillae, distributed as 5 precloacal + 1–2 adcloacal + 7–8 postcloacal (Figure 2A; Table 1).

**Table 1:** Morphometric data of *Ascaridia hermaphrodita* (Froelich, 1789) and *Ascaridia columbae* (Gmelin, 1790) collected from the intestines of captive psittacines in Brazil.

**Character**	***Ascaridia hermaphrodita***	***Ascaridia columbae***
**Males**		
Length	39,442.3 ± 2,671.2 (35,475.0 - 42,683.8)	16,271.0 ± 2,699.8 (13,586.6 - 21,210.2)
Maximum width	1,287.8 ± 151.6 (1,109.8 - 1,562.7)	542.9 ± 84.4 (417.7 - 677.0)
Lip length	142.9 ± 21.7 (103.5 - 167.4)	87.1 ± 12.0 (70.0 - 103.4)
Esophagus length	2,781.5 ± 214.0 (2,581.0 - 3,041.9)	1,363.1 ± 167.9 (1,077.8 - 1,633.9)
Precloacal sucker diameter	270.7 ± 20.1 (240.8 - 303.2)	192.5 ± 21.2 (160.4 - 218.6)
Distance from sucker to cloaca	403.1 ± 38.3 (347.2 - 445.5)	223.1 ± 29.4 (154.0 - 246.8)
Tail length	583.0 ± 60.0 (522.1 - 666.0)	406.7 ± 60.0 (355.3 - 523.8)
Spicule 1 length	2,467.2 ± 221.2 (2,268.7 - 2,822.9)	1,019.9 ± 157.4 (821.3 - 1,266.0)
Spicule 2 length	2,317.9 ± 54.0 (2,253.0 - 2,396.1)	984.0 ± 160.8 (809.6 - 1,185.1)
**Females**		
Length	44,150.1 ± 4,567.0 (35,054.4 - 47,507.3)	19,587.4 ± 2,532.0 (16,876.6 - 23,476.4)
Maximum width	1,400.5 ± 103.3 (1,266.4 - 1,534.9)	711.4 ± 171.6 (516.5 - 929.0)
Lip length	161.4 ± 22.7 (128.4 - 187.9)	93.9 ± 25.7 (58.9 - 127.2)
Esophagus length	2,837.8 ± 214.8 (2,481.4 - 3,077.5)	1,631.2 ± 244.5 (1,433.2 - 1,951.2)
Vulva to anterior end	23,401.8 ± 2,324.0 (19,934.0 - 26,553.7)	9,480.0 ± 1,377.9 (7,989.9 - 11,332.2)
Tail length	1,460.4 ± 175.9 (1,157.9 - 1,696.4)	912.9 ± 224.8 (681.5 - 1,212.5)
Egg length	76.1 ± 7.6 (61.1 - 89.7)	71.5 ± 6.9 (62.2 - 91.0)
Egg width	53.4 ± 3.3 (48.1 - 61.7)	70.7 ± 5.7 (60.8 - 83.9)

The values presented are means, standard deviations, and maximum and minimum values in parentheses. All measurements are in micrometers.

*Ascaridia columbae* has a cylindrical body with tapered ends, a mouth with three lips, without interlabia. Their esophagus is cylindrical, club shaped, and without a posterior bulb. Generally, two lateral membranes are present in the esophageal region. Females *A. columbae* have a vulva located just above the middle of the body, and a divergent uterus full of unembrionated eggs with thick shells. The anus is close to the posterior region. Males have feeble caudal wings; spicules equal or subequal, without gubernaculum. Males also have a preanal sucker that is slightly projecting and rounded, with a horny ring. In the posterior region, thirteen pairs of relatively large caudal papillae (distributed as 5 precloacal + 8 postcloacal papillae) were present in *A. columbae* (Figure 2B; Table 1).

## Discussion

This study identified *A. hermaphrodita* in *A. macao*, *A. aestiva*, and *A. vinacea*, while *A. columbae* was found in *P. maximiliani*. Before necropsy, no clinical manifestations were observed in any of these birds by the staff at the places of origin. Unfortunately, due to the high density of animals and the high demand at establishments, the detection of clinical manifestations, often subtle, in affected animals is sometimes missed. As a result, important signs are overlooked, and control measures are not implemented in time.

All birds were kept in outdoor screened enclosures with access to the ground, and in the case of the macaw, it had access to grass, predisposing them to ascariasis infection (Greiner & Ritchie, 1994). *Ara macao* was the only bird found dead in its enclosure, and it is the only case in this study that parasitism may be the cause of death, since during necropsy, parasites were found obstructing some segments of its intestine (Melo et al., 2013).

*Ascaridia hermaphrodita* is considered one of the most frequent species in Neotropical psittacine birds (Pinto et al., 1993). In Brazil, it has been reported in several native species (Travassos, 1913), mainly *A. aestiva, A. macao, Amazona amazonica* (Linnaeus, 1766), *Aratinga cactorum* (Kuhl, 1820), *Diopsittaca nobilis* (Linnaeus, 1758) (Melo et al., 2013), *Psittacara leucophthalmus* (Müller, 1776), *P. maximiliani, Pyrrhura leucotis* (Kuhl, 1820) (Pinto et al., 1993), *Anodorhynchus hyacinthinus* (Latham, 1790) (Barros et al., 2002), and *Amazona farinosa* (Boddaert, 1783) (Lima et al., 2021).

*Pionus maximiliani* infected with *A. columbae* was kept together in the same enclosure as *A. vinacea*, where synanthropic animals such as Columbiformes were observed entering through the screen. Most likely, contact with pigeons is the main route of entry for parasites. Additionally, the enclosure kept 30 other birds of different species and origins, leading to high levels of stress and population density. This environment fosters increased transmission and susceptibility to infections (Siqueira et al., 2017). Furthermore, these factors, when combined with the presence of two different parasite species, increase the risk of coinfection.

*Ascaridia columbae* is primarily found in Columbiformes (Wehr & Hwang, 1964), although it has been reported in some psittacine birds. In Australia, Mines & Green (1983) reported *A. columbae* parasitizing *Alisterus scapularis* (Lichtenstein, 1818), *Polytelis alexandae* (Gould, 1863), *Agapornis* sp. (Selby, 1836), *Neopsephotus bourkii* (Gould, 1841), and *Melopsittacus undulatus* (Shaw, 1805). However, the only report on Neotropical psittacine species was from Italy by Bernardi et al. (2014), who reported this nematode species in *Ara chloroptera*. In Brazil, reports of this species infecting psittacine birds are scarce, the only previous report was made by Ferolla et al. (1976) in *M. undulatus,* an exotic species. This is the first report of *A. columbae* in *P. maximiliani*, a Neotropical species.

In contrast to the results obtained in other studies (Wehr & Hwang, 1964; Mines & Green, 1983; Wilson et al., 1999), erratic larvae were not found in any of the samples submitted for histological analysis. The autolysis stage of some tissues may have contributed to these results.

Some studies limit parasite identification to the genus level and do not provide information about the species (Jordano Brito et al., 2021). Certain reasons can lead to this situation, including carcasses that are not submitted for postmortem examination and a lack of detailed inspection of the gastrointestinal tract during necropsies. In addition, inadequate methods of storage, transportation, and parasite identification prevent the precise determination of species, which primarily rely on macroscopic observation.

Simple coproparasitological examination in search of parasite eggs is used routinely (Ayres et al., 2016). However, given the similarity of eggs from some species, this method can be inaccurate. Species identification is based on parasite morphological characterization. Methods such as clarification with lactophenol and scanning electron microscopy, as used in this report, are valuable (Melo et al., 2013; Salem et al., 2022). Additionally, molecular techniques such as polymerase chain reaction (PCR) and genetic sequencing are valuable tools for species identification, although cost may present a limiting factor (Abe et al., 2015).

Preventive and control strategies must be implemented to reduce the risk of infection. Measures should include avoiding contact with synanthropic animals, maintaining hygiene of enclosures, food and water, and keeping birds of different species and ages in separate enclosures. Furthermore, contact with the ground should be avoided, and floor types that are easier to clean should be implemented. In rehabilitation centers, regular preventive exams and deworming to control infections are essential, especially considering the significant turnover of animals.

Ultimately, the identification of species of *Ascaridia* in psittacine birds is important because of their impact on avian health and welfare, contributing to improving preventive and control measures in mixed collections.
